# A Tri-Modality Image Fusion Method for Target Delineation of Brain Tumors in Radiotherapy

**DOI:** 10.1371/journal.pone.0112187

**Published:** 2014-11-06

**Authors:** Lu Guo, Shuming Shen, Eleanor Harris, Zheng Wang, Wei Jiang, Yu Guo, Yuanming Feng

**Affiliations:** 1 Department of Biomedical Engineering, Tianjin University, Tianjin, China; 2 Department of Radiation Oncology, East Carolina University, Greenville, North Carolina, United States of America; 3 Department of Radiation Oncology, Tianjin Huanhu Hospital, Tianjin, China; University of California Davis, United States of America

## Abstract

**Purpose:**

To develop a tri-modality image fusion method for better target delineation in image-guided radiotherapy for patients with brain tumors.

**Methods:**

A new method of tri-modality image fusion was developed, which can fuse and display all image sets in one panel and one operation. And a feasibility study in gross tumor volume (GTV) delineation using data from three patients with brain tumors was conducted, which included images of simulation CT, MRI, and ^18^F-fluorodeoxyglucose positron emission tomography (^18^F-FDG PET) examinations before radiotherapy. Tri-modality image fusion was implemented after image registrations of CT+PET and CT+MRI, and the transparency weight of each modality could be adjusted and set by users. Three radiation oncologists delineated GTVs for all patients using dual-modality (MRI/CT) and tri-modality (MRI/CT/PET) image fusion respectively. Inter-observer variation was assessed by the coefficient of variation (COV), the average distance between surface and centroid (ADSC), and the local standard deviation (SD_local_). Analysis of COV was also performed to evaluate intra-observer volume variation.

**Results:**

The inter-observer variation analysis showed that, the mean COV was 0.14(±0.09) and 0.07(±0.01) for dual-modality and tri-modality respectively; the standard deviation of ADSC was significantly reduced (*p*<0.05) with tri-modality; SD_local_ averaged over median GTV surface was reduced in patient 2 (from 0.57 cm to 0.39 cm) and patient 3 (from 0.42 cm to 0.36 cm) with the new method. The intra-observer volume variation was also significantly reduced (*p* = 0.00) with the tri-modality method as compared with using the dual-modality method.

**Conclusion:**

With the new tri-modality image fusion method smaller inter- and intra-observer variation in GTV definition for the brain tumors can be achieved, which improves the consistency and accuracy for target delineation in individualized radiotherapy.

## Introduction

Accurate target definition plays a crucial role in radiotherapy planning of brain tumors, especially, in the image guided radiotherapy (IGRT) which aims at reducing treatment volume toward target volume while ensuring coverage of target volume in all dimensions [1]. To avoid missing target, the intracranial cancerous tissue involvement must be correctly defined in the gross tumor volume (GTV) delineation. Oftentimes single imaging modality cannot provide sufficient information because of its inherent limitations of discriminating different brain soft-tissues or diseased tissues, and combination of different imaging modalities has to be utilized to get more comprehensive understanding of the disease and fulfill an accurate GTV delineation. Therefore, a proper integration of multiple information resources is necessary for the definition of intracranial tumor extension.

Studies showed that utilization of functional imaging techniques, such as ^18^F-fluorodeoxyglucose positron emission tomography (^18^F-FDG PET), ^18^F-fluoromisonidazole PET (^18^F-FMISO PET), diffusion-weighted MRI (DW-MRI) and dynamic contrast-enhanced MRI (DCE-MRI) in addition to CT provides important value to radiotherapy treatment planning for head and neck squamous cell carcinoma (HNSCC) and early response assessment [2]. And in routine radiotherapy practice delineation of GTV for brain tumors relies upon CT or MRI images, or both. However, there are limitations in visualizing tumor and detecting anaplastic tissue with these two image modalities, which may lead to potential inaccuracy in the lesion definition at different steps of the brain tumor management [3]. For example, the conventional MRI is inefficient for the visualization of gliomatous tissue after therapy [4]. Interpretation of anatomical and functional information from multimodal images opens up new possibilities for optimization of the brain tumor treatment. It was shown that PET/MRI coregistration can significantly improve the sensitivity and specificity of ^18^F-FDG in evaluating recurrent tumor and treatment-induced changes for brain tumor [5]. And the radiosurgery guided by the combination of MRI or CT and PET stereotactic images was proved to be valuable for treatment [6]. The GTV could be outlined with greater accuracy with the utility of CT, MRI and PET image fusion technique for target delineation in resected high-grade gliomas before radiotherapy [7]. Furthermore, Grosu et al showed that the composite CT, MRI and PET volumes could improve target volume definition and might further reduce inter-observer variability in stereotactic fractionated radiotherapy treatment planning for meningiomas and gliomas [8], [9].

The dual-modality image fusion techniques provided in current radiotherapy treatment planning systems (TPS) can fuse and display two image sets in one panel and in one operation. And two fused image pairs will be displayed in two separate panels when using three image sets of different modalities, which is inconvenient for target definition.

A commercial system named Advantage Workstation (GE Healthcare, Milwaukee, WI) is recently available in radiology which can provide PET/CT + MR tri-modality image fusion in one application for optimized brain and body imaging. Although three image sets have been obtained, it displays at most two sets in one panel [10]. And there are not yet validation reports about this tri-modality image fusion technique for radiotherapy treatment planning of patients with brain tumors.

We recently developed a new tri-modality image fusion method which can fuse and display all image sets in one panel and one operation. Its effectiveness for brain tumor GTV delineation was quantitatively evaluated through comparative study with using the current dual-modality image fusion techniques. The method and feasibility study are presented in this report.

## Materials and Methods

### 1. Image data acquisition

Image data of simulation CT, MRI, and ^18^F-FDG PET-CT prior to radiation therapy from three patients were used in this study with the patients’ written consents. The study and consent procedure were specifically approved by the ethics committee of Tianjin Huanhu Hospital and the authors had explicit permission to publish the patient brain images. Among the three patients, one (male, 69 years old) had high-grade glioma and two (one female and one male, 71 and 47 years old, respectively.) had brain metastasis tumors from their non-small cell lung cancer and thyroid carcinoma, respectively. None of these lesions had previously been resected.

CT image sets for radiation treatment planning for the patients was acquired using a Brilliance Big Bore CT (Philips Medical Systems) in supine and head first position. Total number of transverse slices was 224 with slice thickness of 2 mm, and field of view of 242×242 mm^2^ and 224×224 mm^2^ for the patient with gliomas and brain metastasis respectively.

PET-CT data were obtained with a Biograph64 PET-CT scanner (Siemens Medical Solutions). The applied dose of ^18^F-FDG was between 246 and 296 MBq for the imaging studies. The patients were positioned supine with head first and 75 transverse images were acquired from a transmission scan of 3 and 4 min duration time for the patient with gliomas and brain metastasis respectively. The slice thickness was 3 mm, and axial field of view was 407×407 mm^2^.

MRI data were obtained with a Magnetom Trio Tim scanner (Siemens Medical Solutions). With contrast medium of gadolinium-diethylenetriaminepentacetic acid [Gd-DTPA] (0.1 mmol/kg body weight), the axial, gradient echo T1-weighted sequences were acquired from the foramen magnum to the vertex (repetition time = 250 ms, echo time = 2.46 ms, imaging frequency = 123. 2 Hz, magnetic field strength = 3T, flip angle = 70°). The slice thickness was 2 mm, and field of view was 230×230 mm^2^.

### 2. Image registration

Normally, the rigid bone of skull confines the brain tissue and leads to little significant non-rigid transformation between the different image sets. Therefore, three-dimensional (3D) rigid-body registration is sufficient for aligning those head image sets from different modalities to the same coordinate system. The rigid-body registrations of CT+PET and CT+MRI were completed respectively with the automatic image registration tool of MIM 5.2 (MIM Software Inc., Cleveland, OH). Based on the mutual information measure, the software mapped the secondary image sets of PET and MRI to the primary image set of CT respectively. Subsequently, these aligned secondary image sets were imported into an in-house developed software for tri-modality image fusion.

### 3. New algorithm for tri-modality image fusion

**Figure 1:**
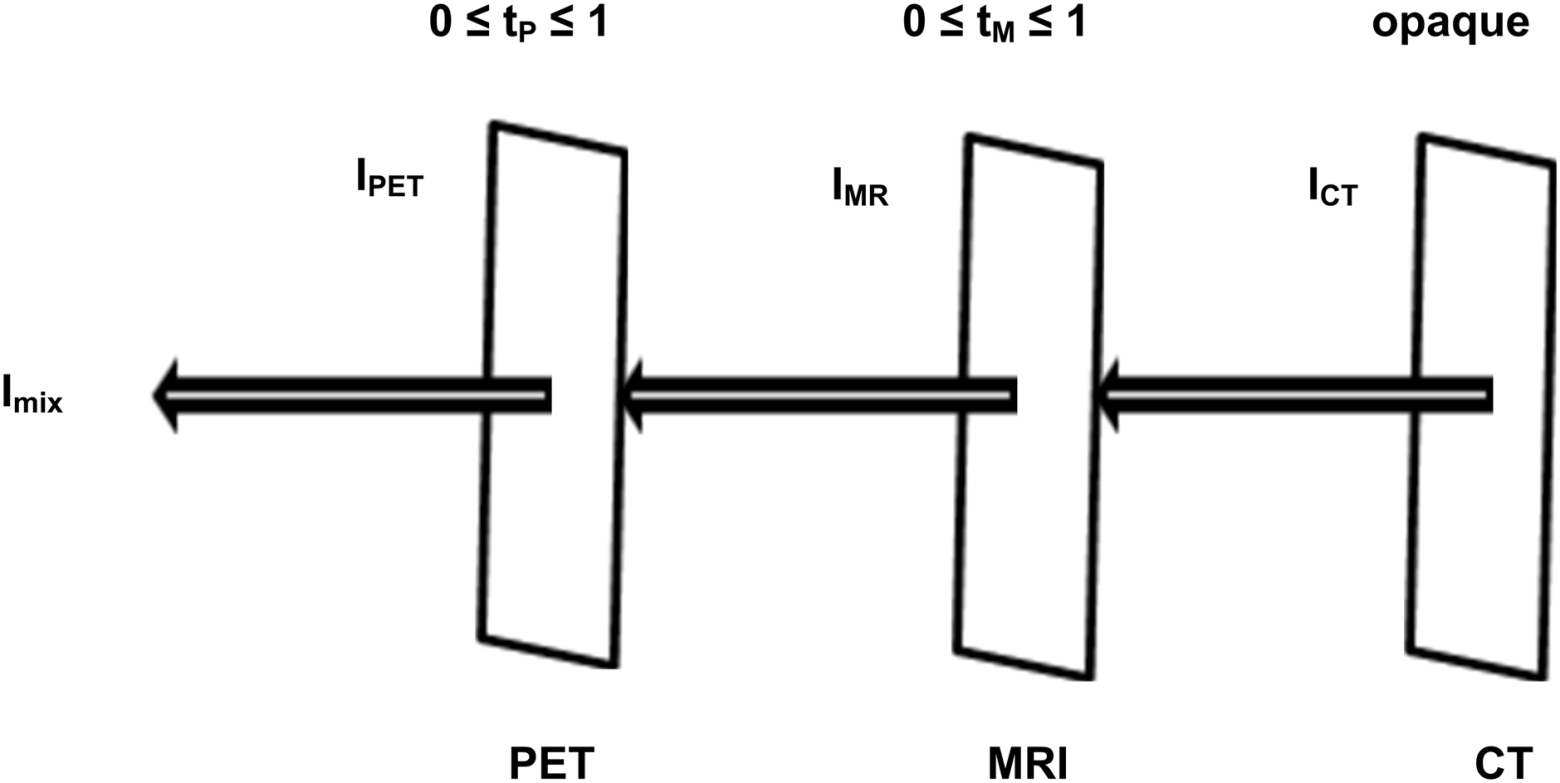
Illustration of tri-modality image fusion. The intensity value of a pixel in the fused image (I_mix_) is determined based on the corresponding pixel’s intensity values in CT, MRI, PET images (I_CT_, I_MRI_, I_PET_).

**Figure 2:**
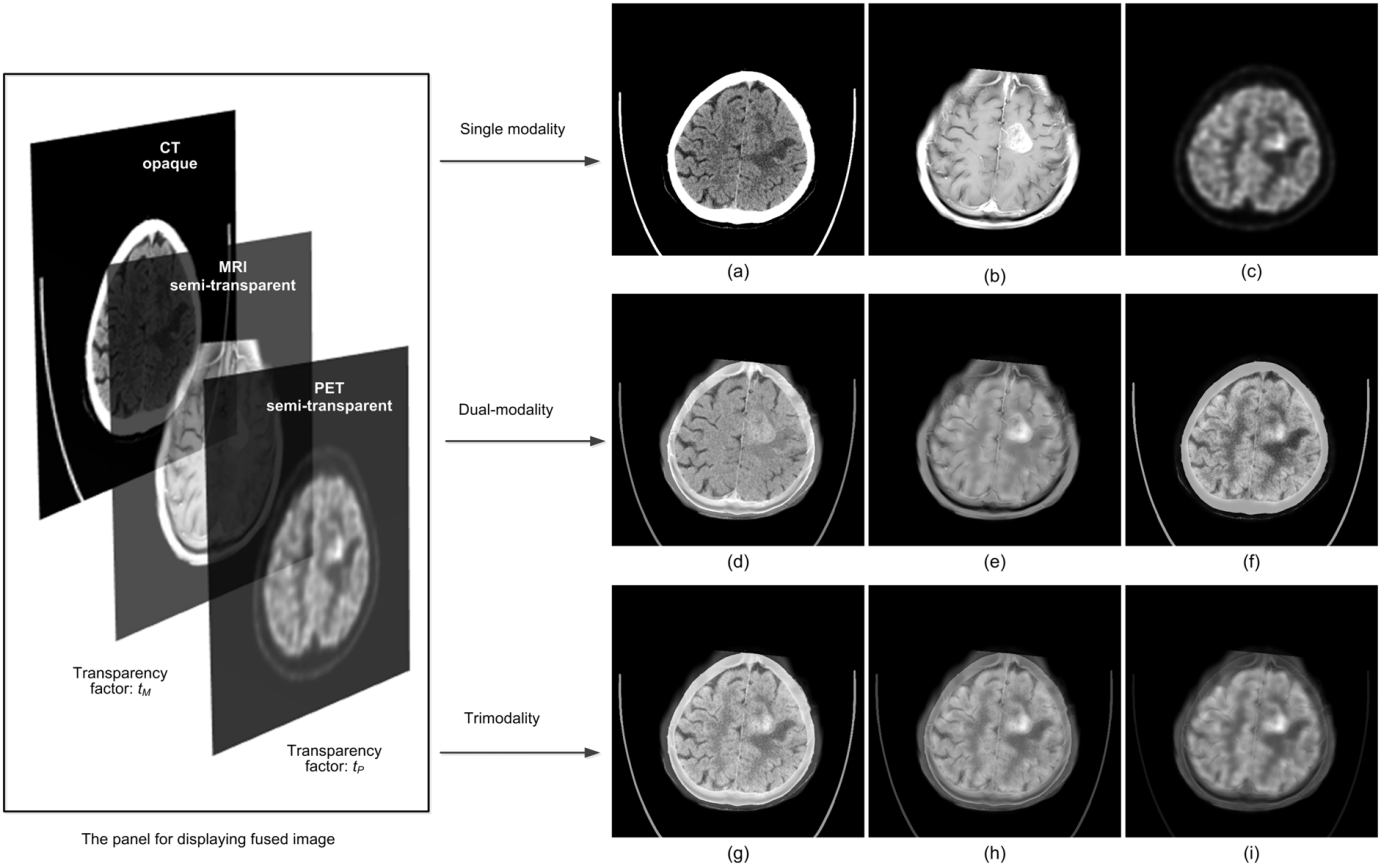
A glioma tumor shown on CT, MRI, and PET images with various *t_P_* and *t_M_* altering in the range of 0 to 1 for patient 1. (a) t_P_ = 0, t_M_ = 0; (b) t_P_ = 0, t_M_ = 1;(c) t_P_ = 1, t_M_ = 0; (d) t_P_ = 0, t_M_ = 0.5; (e) t_P_ = 0.5, t_M_ = 1;(f) t_P_ = 0.5, t_M_ = 0; (g) t_P_ = 0.3, t_M_ = 0.3; (h) t_P_ = 0.5, t_M_ = 0.5; (i) t_P_ = 0.7, t_M_ = 0.7.

In the study, we set CT image as the primary, background opaque surface and PET/MR images as the foreground semi-transparent surfaces. Based on the transparency model in computer graphics [11], [12], we proposed a new algorithm for tri-modality medical image fusion which is illustrated in Figure 1 and expressed with Equation 1.
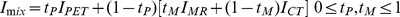
(1)Here *I_mix_* is the intensity value of a pixel in the fused image; *I_PET_*, *I_MR_*, *I_CT_* are the intensity values of the corresponding pixels in PET, MR, and CT images respectively; *t_P_* and *t_M_* are the transparency factor of PET and MR images, respectively, which can be adjusted by the user according to the desired transparency weight of each modality. The display of the fused image would vary along with the change of transparency factor (*t_P_*, *t_M_*). It provides a convenient tool for the user to look for target information over composite images of single modality, dual-modality, or tri-modality in one single panel (Figure 2).

### 4. GTV delineation

The same group of three radiation oncologists (observers) performed target delineation independently on the composite images of dual-modality image fusion (MRI/CT) and of tri-modality image fusion (MRI/CT/PET), respectively, using the contouring protocol published by Graf et al [13]. The GTVs were first contoured with CT and MRI image sets only and then corrected or re-delineated using the information from PET images. GTV delineation on CT, MRI, and PET was performed according to the following steps: (1) The contrast enhanced T1-weighted MRI images were used to define GTV_MRI_. (2) CT images were adjusted to bone window and GTV_MRI_ was expand to enclose all tumor manifestation in CT or MRI images to obtain the GTV_MRI/CT_, which was the tumor volume defined with dual-modality image fusion (CT/MRI). (3) Then, the GTV_PET_ was delineated on ^18^F-FDG PET image set without referencing the GTV_MRI/CT_. The window setting for ^18^F-FDG-PET was made to optimize alignment between tumor margins on MRI/CT and ^18^F-FDG-PET in area with tumor-to-normal brain tissue interface. (4) The overlapping region of GTV_MRI/CT_ and GTV_PET_ was defined as GTV_common_ which encompassed the region with pathologic changes shown on all three image modalities (CT, MRI and PET image sets). (5) The volume of GTV_MRI/CT_ located outside the region of enhanced 18F-FDG uptake was re-evaluated by the observers based on the information obtained from CT/MRI/PET image fusion. (6) Thereafter, a part of GTV_MRI/CT_ was added to GTV_common_. (7) The volume of GTV_PET_ located outside the region with pathologic changes shown on MRI/CT was re-evaluated in the same way. (8) Similarly, a part of GTV_PET_ was added to the GTV_common_. (9) The final GTV (GTV_MRI/CT/PET_) defined with tri-modality image fusion (CT/MRI/PET) consisted of GTV_common_ and the added parts from GTV_MRI/CT_ and GTV_PET_. Thus, each observer defined two GTVs (GTV_MRI/CT_ and GTV_MRI/CT/PET_) for the same tumor of each patient in the two composite image sets, respectively.

### 5. Evaluation of inter-observer variation

#### 5.1 Volume variation

To evaluate the inter-observer variation in the delineations of the GTVs with the schemes of dual-modality image fusion and tri-modality image fusion described in 2.4, GTV volumes of the tumors obtained by each observer were used and the coefficient of variation (COV) of the three volumes defined by the three observers with each scheme for each tumor were calculated and statistically analyzed [14]. COV is defined as the ratio of the standard deviation (SD) to the mean value. And SPSS Statistics 20.0.0. (SPSS Inc., Chicago, IL) was used for a paired sample two-tailed *t* test on COV to statistically measure the difference between the means for the two schemes. A two-side *p* value of less than 0.05 was considered to be significant.

#### 5.2 Shape variation

A 3D shape was generated using the marching cubes (MC) algorithm [15] for each GTV involved in this inter-observer variation evaluation. Subsequently, the average distance between surface and centroid (ADSC) was calculated by averaging the distance of each triangle on the surface to its centroid. For each patient and each scheme, the mean and SD of ADSC of the three observers were calculated as a measurement of the inter-observer shape variation. The paired sample two-tailed *t* test was applied to evaluate the difference between using dual- and tri-modality image fusion methods.

Furthermore, a 3D median surface of GTVs delineated by the three observers was computed using Deurloo’s method [16], [17] for each patient and each scheme. The median surface was equal to the 50% coverage probability matrix of the three GTVs; in other words, all points in the median surface were determined at least by 50% observers. Afterwards, we calculated the perpendicular distance along the normal vector from each vertex point on the median surface to each GTV surface delineated by the observers. Three distances were obtained for each vertex point constructing the median surface. The standard variation of the three distances represents the local variation on the 3D surface and is called “local standard deviation (SD_local_)” which shows the level of variation in contouring details of GTVs. Subsequently, the median surface was divided into eight regions based on the eight quadrants of a new 3D coordinate system which moved the original point to the mean centroid of three individual GTVs. SD_local_ was averaged at eight regions and all vertex points on the median surface for each patient and each scheme.

### 6. Evaluation of intra-observer volume variation

The intra-observer variation in GTV volume definition was also investigated. Each GTV was contoured three times by the three observers, respectively. The averaged time interval between these three delineations was about two months. Totally, 54 GTVs were obtained for the analysis. Mean values and SDs of the GTV volumes and the COV were calculated, and the intra-observer variation was statistically analyzed.

## Results

### 1. Inter-observer variation of GTV volumes

The volumes of GTVs for each patient defined by each observer, the mean values of the GTV volumes from the three observers, and the COV with the two schemes are summarized in Table 1. It can be seen that the volumes of GTVs defined with the first scheme varies more than the ones with the second scheme. All the mean values of GTV volumes defined by the three observers are reduced with the second scheme. The mean COV is 0.14(±0.09) and 0.07(±0.01) for the first and second scheme, respectively, but there is no significant difference statistically (*p* = 0.30>0.05, 95% confidence interval of the difference is [–0.15, 0.31]), revealing that with the tri-modality image fusion the inter-observer volume variation is not significantly reduced.

**Table 1:** Comparison of GTV volumes defined by each observer with dual-modality image fusion (MRI/CT) and tri-modality (MRI/CT/PET) image fusion.

Patient	GTV_MRI/CT_ (cm^3^)					GTV_MRI/CT/PET_ (cm^3^)				
	Obs. 1	Obs. 2	Obs. 3	Mean	COV	Obs. 1	Obs. 2	Obs. 3	Mean	COV
**1**	19.91	22.72	20.47	21.03	0.07	17.91	19.82	17.69	18.47	0.06
**2**	14.39	17.01	13.94	15.11	0.11	12.61	14.09	13.27	13.32	0.06
**3**	2.96	2.92	1.82	2.57	0.25	1.89	1.92	1.64	1.82	0.08

GTV = gross tumor volume; Obs. 1 = observer 1; COV = coefficient of variation.

### 2. Inter-observer variation of GTV shapes

The comparison of ADSC between the first and second scheme for each patient and observer is shown in Table 2. For all three cases, the means of ADSC with the second scheme are smaller than the ones with the first scheme. The SDs of ADSC is largely reduced with the second scheme in all cases. And the paired sample two-tailed *t* test shows that the reduction is statistically significant (*p*<0.05, 95% confidence interval of the difference is [0.01, 0.05]), demonstrating that 3D shape variation between the GTVs defined by the three observers is significantly reduced with the new tri-modality image fusion method.

**Table 2:** Comparison of ADSC (in cm) for each patient and each observer using dual-modality (MRI/CT) fusion vs. tri-modality (MRI/CT/PET) fusion.

Patient	ADSC with Dual-modality					ADSC with Tri-modality				
	Obs. 1	Obs. 2	Obs. 3	Mean	SD	Obs. 1	Obs. 2	Obs. 3	Mean	SD
**1**	1.77	1.86	1.79	1.81	0.05	1.73	1.77	1.72	1.74	0.03
**2**	1.58	1.67	1.58	1.61	0.05	1.51	1.56	1.53	1.53	0.03
**3**	0.95	0.97	0.85	0.92	0.06	0.83	0.84	0.82	0.83	0.01

ADSC = the average distance between surface and centroid; Obs. 1 = observer 1; SD = standard deviation.

The SD_local_ was averaged at eight quadrants and all vertex points on the median surface for each patient and each scheme (Table 3). For patient 1, SD_local_ is partly reduced with the second scheme at quadrant No. 1 and No. 4. For patient 2, there are large reductions of SD_local_ at seven quadrants, especially at No. 3 (ΔSD_local_ = 0.5 cm), No. 4 (ΔSD_local_ = 0.25 cm), and No. 7 (ΔSD_local_ = 0.25 cm). For patient 3, it is at six quadrants that the SD_local_ is reduced, especially at No. 5 (ΔSD_local_ = 0.21 cm).

**Table 3:** SD_local_ (in cm) of GTV shape variation relative to the median GTV shape for each patient at eight quadrants and average over all vertex points on the median GTV.

Modality	Patient	Quadrants No.								Averageover GTV
		1	2	3	4	5	6	7	8	
**Dual-** **modality**	**Patient 1**	0.38	0.39	0.56	0.37	0.47	0.45	0.47	0.42	0.44
	**Patient 2**	0.31	0.46	0.89	0.60	0.42	0.39	0.91	0.56	0.57
	**Patient 3**	0.50	0.41	0.32	0.39	0.69	0.42	0.27	0.30	0.42
**Tri-** **modality**	**Patient 1**	0.36	0.46	0.71	0.33	0.53	0.47	0.55	0.48	0.50
	**Patient 2**	0.32	0.35	0.39	0.35	0.33	0.34	0.66	0.44	0.39
	**Patient 3**	0.37	0.29	0.27	0.35	0.48	0.35	0.38	0.41	0.36

SD_local_ = local standard deviation; GTV = gross tumor volume.

**Figure 3:**
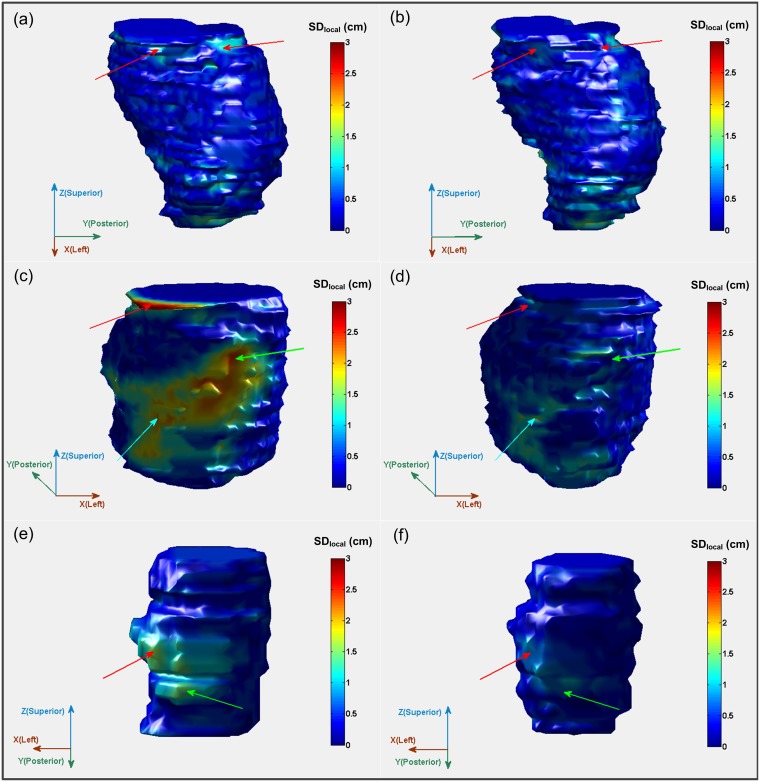
Comparison of the local standard deviation (SD_local_) mapped onto the median surface of GTVs with dual-modality fusion (a, c, e) and GTVs with tri-modality fusion (b, d, f) in color wash (from blue [SD_local_≤0.1 cm] to red [SD_local_≥3 cm]). Red arrows in (a) and (b) point to the same region where SD_local_ is partly reduced with the second scheme for patient 1. Red, green and light blue arrows in (c) and (d) point to the same regions of three quadrants where SD_local_ is largely reduced with the second scheme for patient 2 (ΔSD_local_≥0.2 cm). Red and green arrows in (e) and (f) point to the same regions of two quadrants where SD_local_ is largely reduced with the second scheme for patient 3 (ΔSD_local_≥0.2 cm in the area pointed by red arrows).

The median surfaces of GTVs onto which the SD_local_ was mapped in color for three patients are shown in Figure 3. Up to about 1 cm in SD_local_ has been observed with both schemes (Figure 3a and 3b) for patient 1. Whereas, there are two regions on which the SD_local_ is reduced obviously with the second scheme (the areas pointed with red arrows in Figure 3b). For patient 2, SD_local_ in a large part of areas (quadrant No. 3, No. 4, and No. 7 as indicated by red, green and light blue arrows respectively in Figure 3c) on the median surface of GTVs from the first scheme is larger than 1 cm and the maximum is larger than 3 cm. For the median surface of GTVs from the second scheme (Figure 3d), however, the maximum SD_local_ is 1.64 cm, much smaller than the former. For patient 3, up to 1.5 cm in SD_local_ is observed in two quadrants of the median surface of GTVs from the first scheme (Figure 3e) while the maximum SD_local_ of 1.15 cm over all vertex points on median surface is observed for the GTVs from the second scheme (Figure 3f).

**Figure 4:**
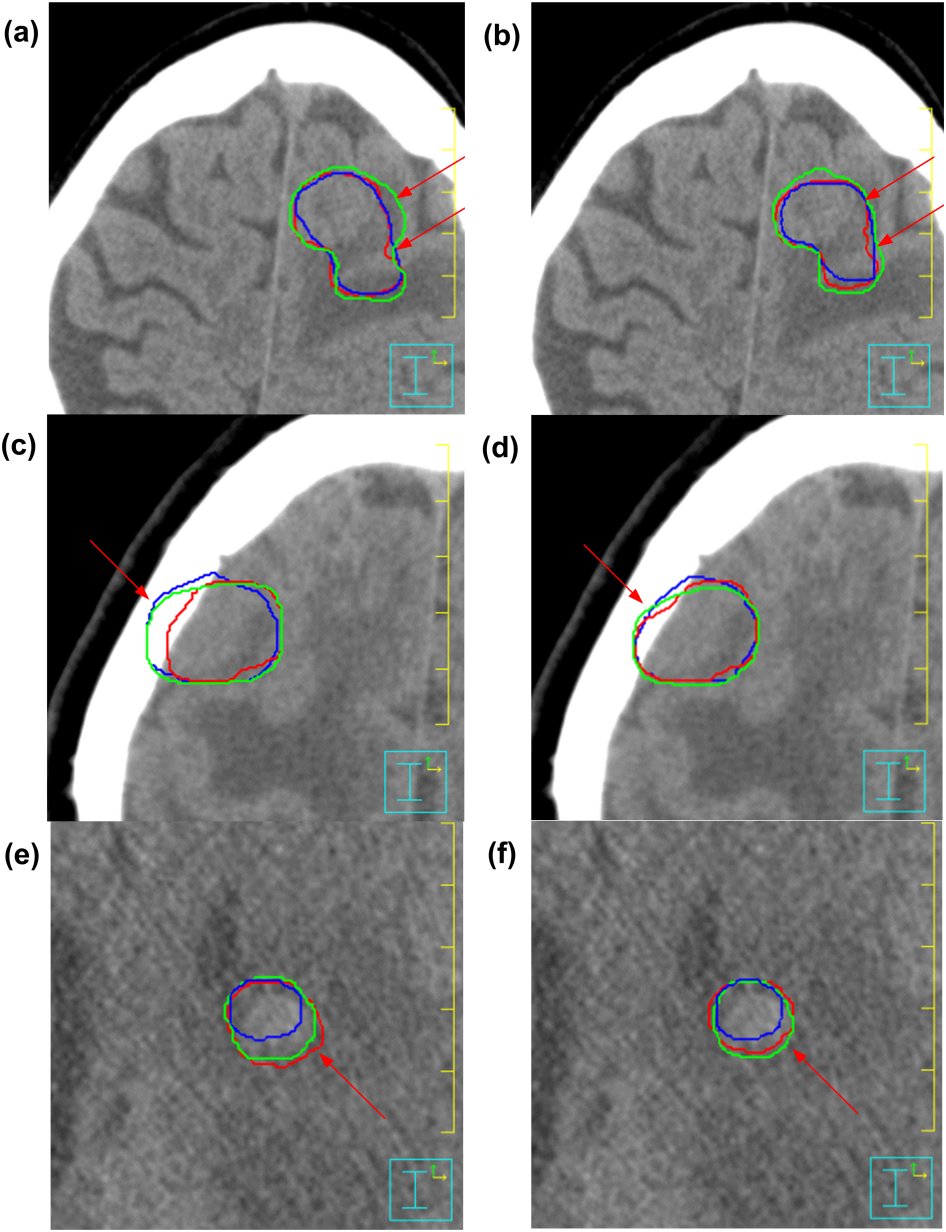
The GTVs delineated on axial slices of patient CT images by the three observers with dual-modality fusion (a, c, e) and tri-modality image fusion (b, d, f) methods, respectively. (a) and (b) are for patient 1; (c) and (d) are for patient 2; (e) and (f) are for patient 3.

Large reductions of inter-observer contouring variation in GTV delineation are also observed on axial slices of three patient CT images with the assistance of tri-modality image fusion as shown in Figure 4. The axial slices correspond to the same area in median surface as marked by the red arrows in Figure 3.

### 3. Intra-observer variation of GTV volumes

The mean value and SD of the three repeated GTVs determined by each observer for each patient, the COV with the two schemes are summarized in Table 4. Much smaller SD and COV values are observed with the second scheme than the ones with the first scheme in the results of all three observers’ delineation for the patients. The mean of COV for all three observers is significantly reduced, from 0.05 (±0.03) with the first scheme to 0.02 (±0.01) with the second scheme (*p* = 0.00, 95% confidence interval of the difference is [0.01, 0.06]), revealing that the intra-observer volume variation is significantly reduced with the new tri-modality image fusion method.

**Table 4:** Intra-observer comparison of GTV volumes obtained with dual-modality image fusion (MRI/CT) and tri-modality (MRI/CT/PET) image fusion.

Observer	Patient	GTV with Dual-modality		GTV with Tri-modality	
		Mean ± SD (cm^3^)	COV	Mean ± SD (cm^3^)	COV
**Observer 1**	**Patient 1**	21.52±1.48	0.07	17.15±0.68	0.04
	**Patient 2**	14.05±0.34	0.02	12.44±0.16	0.01
	**Patient 3**	2.64±0.29	0.11	1.87±0.02	0.01
**Observer 2**	**Patient 1**	22.52±1.00	0.04	19.76±0.31	0.02
	**Patient 2**	16.46±0.51	0.03	14.08±0.13	0.01
	**Patient 3**	2.79±0.12	0.04	1.94±0.02	0.01
**Observer 3**	**Patient 1**	20.28±0.96	0.05	17.55±0.47	0.03
	**Patient 2**	14.63±0.60	0.04	13.40±0.17	0.01
	**Patient 3**	1.99±0.16	0.08	1.66±0.02	0.01

GTV = gross tumor volume; COV = coefficient of variation; SD = standard deviation.

## Discussion

Our study has shown that the tri-modality image fusion method which integrates CT, MRI and ^18^F-FDG-PET has positive impact on the radiotherapy treatment planning for brain tumors. The volume of GTV contoured for high-grade gliomas and brain metastasis can be reduced as compared with using dual-modality (CT/MRI) image fusion. The inter-observer variation of GTV shapes (Figure 3, Figure 4 and Table 2) and the intra-observer variation in defining GTV volumes (Table 4) can be significantly reduced.

The interpretation differences between observers, derived from clinical experience, image interpretation skills, compliance to set guidelines, and treatment philosophy [14], were the major component of inter-observer variation in this study. The volume of GTV defined by observer 2 was larger than other two observers’ for each patient and scheme (Table 1). The same trend could be found in Table 2 for the parameter of ADSC. Although variation in target definition existed among the observers even with the tri-modality method, the SDs of ADSC were significantly reduced as compared with using dual-modality method. These results indicate that contours can be drawn more consistently with the new tri-modality image fusion method and large reduction of the inter-observer difference can be achieved in GTV definition for brain tumors.

The new method provides convenience to observers in the operation for target delineation due to the adjustable transparency values of foreground images in one single panel, which has not been reported in published studies so far to our knowledge. With our in-house developed software tool, observers are able to view composite images in single modality, dual-modality, or tri-modality mode in one single panel. Observers can change the transparency factors (*t_P_*, *t_M_*) and gain various combinations of tri-modality images’ information to facilitate a more comprehensive definition of final GTV_MRI/CT/PET_. And the lessened intra-observer variation of GTV volumes (mean COV is 0.05 and 0.02 for dual- and tri-modality image fusion, respectively) in our study manifests the high reproducibility of results with this software.

Although a relatively small amount of raw data was used in this feasibility study because of the difficulty of getting image data of three modalities for the same tumor site, the results have shown potentials for utilizing three modality image data for the cases for which two image modalities cannot provide sufficient information in target definition for the brain tumors. When the tumor locates close to a critical normal structure or tissue, this method will be helpful for reducing uncertainty in tumor boundary definition and sparing the adjacent normal tissues, especially when high fractional dose and small margins around the tumor are applied.

## Conclusion

With the tri-modality (CT/MRI/PET) image fusion method, the inter-observer variation in the delineation of GTV shapes and intra-observer variation in GTV volumes definition for the brain tumors can be reduced. This finding reveals the potential usability of this method for reducing uncertainty in brain tumor boundary delineation in radiotherapy treatment planning.
